# The effect of hydroxy‐selenomethionine on the productive and reproductive performance of old broiler breeders

**DOI:** 10.1002/vms3.1538

**Published:** 2024-07-10

**Authors:** Ahmad Manafi, Yahya Ebrahimnezhad, Habib Aghdam Shahryar, Abdolahad Shaddel Teli, Abolfazl Gorbani, Naser Maheri‐Sis

**Affiliations:** ^1^ Department of Animal Science, Shabestar Branch Islamic Azad University Shabestar Iran

**Keywords:** broiler breeder roosters, broiler breeders, hatchability, hydroxy‐selenomethionine, sperm quality parameters

## Abstract

**Background:**

Selenium (Se) is a rare essential element that plays a vital role in the health and performance of animals. By interfering in the production of antioxidant enzymes such as glutathione peroxidase, thioredoxin reductase and methionine sulfoxide, Se plays a role in reducing the effects of oxidative stress and animal performance.

**Objectives:**

This study aimed to investigate the effect of hydroxy‐selenomethionine (OH‐SeMet) in the diet of broiler breeder and old broiler breeder roosters on productive performance, reproduction and sperm quality parameters.

**Methods:**

For this purpose, 260 broiler breeders of the Ross 308 strain were used in a completely randomized design with four treatments and five replications (13 hens and one rooster in each replication). Experimental treatments included: (1) a basal diet without OH‐SeMet (T1:control), (2) a broiler breeder diet without OH‐SeMet and a rooster diet containing 0.1 mg/kg OH‐SeMet (T2), (3) broiler breeder diet containing 0.1 mg/kg OH‐SeMet and rooster diet without OH‐SeMet (T3) and (4) broiler breeder and rooster diet contained 0.1 mg/kg OH‐SeMet (T4).

**Results:**

The results showed that T3 and T4 treatments improved egg production, egg weight, egg mass and feed conversion ratio (FCR) compared to the control treatment (*p* < 0.05). The fertility and hatchability percentages of T4 and T2 treatments increased compared to T1 and T3 treatments (*p* < 0.05). The rate of embryonic losses in T1 was higher than in other treatments. However, grade one chickens were higher in T4 than in other treatments (*p* < 0.05). Total motility and viability of sperms were significantly higher in T2 and T4 treatments than in T1 and T3 treatments. The sperm abnormality percentage and sperm MDA concentration decreased in T2 and T4 treatments.

**Conclusions:**

Therefore, using OH‐SeMet may be a practical approach to help old broiler breeders’ production and reproduction performance.

## Graphical abstract

The use of 0.1 mg/kg hydroxy‐selenomethionine in broiler breeders’ diet improved egg production, egg weight, feed efficiency, hatchability, fertility and progeny quality. Moreover, this supplement improved sperm quality parameters in old roosters, including total motility, viability, abnormality and MDA. The results show the better potential of this form of selenium with a lower dose than the inorganic form in broiler breeders’ diet, which requires further research on types of forms and concentrations.

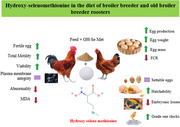

## 1 INTRODUCTION

Selenium (Se) is a rare essential element that plays a vital role in the health and performance of animals. Se deficiency can have important negative effects from an economic point of view, such as reducing the performance and fertility of animals (Spears & Weiss, 2008). By interfering with the production of antioxidant enzymes such as glutathione peroxidase, thioredoxin reductase and methionine sulfoxide, Se plays a role in reducing the effects of oxidative stress and improving animal health (Bakhshalinejad et al., 2018). When it comes to broiler breeders’ diets, there are two primary factors to take into account regarding Se. Firstly, Se is known to be crucial for preserving the quality of semen, and a rooster's optimal Se status is thought to be a key component in ensuring fertility (Surai, 2018). Secondly, the antioxidant system of the developing embryo depends critically on the Se content of the eggs produced by broiler breeders. Enhancing the embryo's antioxidant defense can potentially increase hatchability as the incubation process has been widely shown to causes oxidative stress (Rajashree et al., 2014). Li et al. (2020) observed that adding 0.15 mg/kg of selenomethionine to the diets of broiler breeders reduced embryo mortality under oxidative stress brought on by the diquat challenge. Stressful environments cause oxidative stress in poultry, which has negative effects on health (immune system suppression, increased inflammatory response), reproduction (lower fertility and hatching), growth and FCR (Surai, 2018; Bottje, 2019; Pappas et al., 2006). Oxidative stress occurs when the production of free radicals' surpasses the antioxidant systems’ protective ability. Furthermore, when diets are lacking in Se or when Se requirements are not fully met, broiler breeders and broilers may decline in overall performance (Zhao et al., 2019).

Commercial chicken diets are always supplemented with Se through premixes of 0.2 to 0.3 mg/kg, depending on the source, to prevent its deficiency since many feed ingredients are low in minerals. In poultry diets, Se can be utilized in organic forms, such as Se‐fermented yeasts, inorganic forms, like sodium selenite or pure chemical forms, like hydroxy‐selenomethionine (OH‐SeMet) or L‐selenomethionine. The tendency to use organic Se in the diet has increased due to the unfavorable characteristics of its inorganic form, such as low efficiency and bioavailability, toxicity, interactions with other minerals and inability to maintain body reserves of Se (Han et al., 2009). Thorough research has been done on the advantages of organic Se compounds (like selenomethionine) in the diet of broiler breeders. According to Surai and Fisinin (2014), Se organic compounds have been shown to considerably boost the body's Se reserves in birds by causing selenomethionine to deposit, which improves the birds’ antioxidant defenses.

Furthermore, organic Se compounds are more effectively transferred from feed to eggs and subsequently to developing embryos compared to inorganic Se (Wang et al., 2021). This is a useful tactic to help chickens strengthen their resistance to a variety of stresses and reset their antioxidant defense system. Additionally, studies have shown that broiler breeders’ diets containing organic compounds high in Se may have positive effects on offspering during the rearing period (Zhang et al., 2014). Recently, a new source of stable organic selenium, which is a hydroxy analogue of selenomethionine, has been created and is used instead os selnomethionine, which is unstable and easily oxidized in each pure form (Pavaneli et al., 2021). The higher bioavailability of seleno‐amino acid composition compared to other organic or inorganic forms has been confirmed previously by Couloigner et al. (2015), reducing the amount of Se supplementation required in the diet.

Fertility is one of the most important features that ensures profitability in the poultry industry, and roosters play a prominent role in this (Ahsan et al., 2014). Roosters reach their maximum fertility at the age of 32–40 weeks, but their fertility decreases around the age of 45 weeks, which can have a negative effect on the profitability of the herds (Qazi et al., 2019). Hence, improving sperm quality in older roosters. Nutrition has a great effect on the quality and quantity of sperm, and therefore, enriching the diet with multi‐That maintain this for longer is curcial to maximizing economic benefits. Nutrition has a great effect on the quality and quantity of sperm, and therefor, enriching the diet with multi‐functional compounds is a solution to deal with the decrease in fertility in old roosters (Alavi et al., 2020). Given that a large amount of polyunsaturated fatty acids (PUFAs), which are susceptible to lipid peroxidation, are present in avian semen, dietary Se is believed to improve the quality of sperm produced (Surai et al., 1998). However, currently, the hydroxy form of Se has become more favourable due to its much higher bioavailability and lower toxicity compared to the inorganic and other organic forms. This is due the unique properties of hydroxy, including high surface activity, active centres, countless surface area, high catalytic efficiency, and strong absorption ability and low toxicity. In addition, since the surface to volume ratio increases with as particle size decreases hydroxy Se has higher biological activity, including its anti‐hydroxyl radical property and protective actions against DNA oxidation (Zorzetto et al., 2021). Therefore, it seems that OH‐SeMet is more effective than the other two forms of Se in preventing the decrease in the performance of broiler breeders and the fertility of old roosters, and thus it needs to be investigated. According to Ebeid (2012), adding organic supplementation (0.3 mg Se/kg diet) to roosters’ diets increased the plasma GSH‐Px activity of semen and semen quality by reducing lipid peroxidation. Recently, Zorzetto et al. (2021) investigated the effect of replacing sodium selenite with a lower level of OH‐SeMet on the performance of broiler breeders and their offspring. Results showed that 0.2 mg OH‐SeMet increased egg production, Se content in eggs, shell strength and hatchability compared to 0.3 mg sodium selenite.

According to the a formentioned materials, using organic Se in feeding broiler breeders and old broiler breeder roosters may improve production and reproductive performance. Therefore, this study aimed to investigate the effect of OH‐SeMet in the diet of broiler breeders and old broiler breeder roosters on productive performance, reproduction and sperm quality parameters.

## 2 MATERIALS AND METHODS

### 2.1 Birds, diets and management

This experiment was conducted using 260 broiler breeders and 20 broiler breeder roosters (45 weeks old) of the Ross 308 strain in a completely randomized design with five replications (13 hens and one rooster per replication) for 90 days. Broiler breeding instructions, including control of exposure, ventilation, drinking water, vaccination and so on, were implemented for all treatments in the same way and based on the standard conditions of the strain (Aviagen, 2018). Experimental treatments included: (1) a basal diet without OH‐SeMet (T1: control), (2) a broiler breeder diet without OH‐SeMet and a rooster diet (Table 1) containing 0.1 mg/kg OH‐SeMet (T2), (3) broiler breeder diet containing 0.1 mg/kg OH‐SeMet and rooster diet without OH‐SeMet (T3) and (4) broiler breeder and rooster diet contained 0.1 mg/kg OH‐SeMet (T4).

**TABLE 1:** Composition of basal diet of broiler breeders and roosters (as‐fed basis).

Ingredient (g/kg)	Broiler breeders	Broiler breeder roosters
Corn grain	625.0	532.7
Soybean meal (44% CP)	165.0	55.0
Wheat grain	40.0	65.0
Barley grain	0.0	50.0
Wheat bran	40.0	234.0
Soybean oil	12.0	8.0
Di‐calcium phosphate	16.4	12.8
Bentonite	7.05	9.55
Calcium carbonate	77.2	15.4
Vitamin premix^a^	2.5	2.5
Mineral premix^b^	2.5	2.5
Salt	2.2	2.0
Sodium bicarbonate	2.2	2.35
Potassium bicarbonate	1.5	1.5
dl‐Methionine	1.8	1.4
l‐Lysine HCL	0.6	0.8
l‐Threonine	0.8	0.8
Choline chloride	1.0	1.0
Antioxidants	0.2	0.2
Toxin binder	1.0	1.0
Anti‐ammonium extra	1.0	1.0
Phytase	0.05	0.05
Total	1000.0	1000.0
Calculated composition		
Metabolizable energy (kcal/kg)	2800	2825
Crude protein (%)	13.52	11.77
Crude fibre (%)	3.02	4.63
Calcium (%)	3.20	0.90
Available *p* (%)	0.34	0.30
Lysine (SID) (%)	0.60	0.43
Methionine (SID) (%)	0.38	0.30
Met + Cys (SID) (%)	0.61	0.48
Threonine (SID) (%)	0.55	0.39
DCAB (mEq/kg)	203.00	200.00

^a^ Vitamin premix provided per kilogram of diet: vitamin A, 15,000 IU; vitamin D3, 5000 IU; vitamin E, 130 IU; vitamin K3, 9 mg; vitamin B1, 6 mg; vitamin B2, 20 mg; vitamin B6, 8 mg; vitamin B9, 5 mg; vitamin B12, 0.07 mg; biotin, 0.6 mg; niacin, 70 mg; pantothenic acid, 25 mg.

^b^ Mineral premix provided per kilogram of diet: Fe, 50 mg; Cu, 16 mg; Mn, 120 mg; Zn, 120 mg; I, 3 mg; Se, 0.3 mg.

Before adjusting the experimental rations, the food materials were analysed according to the Association of Official Analytical Chemists method (1995), and dry matter, crude protein, ether extract, ash, insoluble fibres in neutral detergent, insoluble fibres in acidic detergent, lignin insoluble in acidic detergent and crude fibre were calculated. The feed consumption in the experimental groups was calculated according to the catalogue (Aviagen, 2018) and provided to the birds (162 g from week 45 to 60 per bird). The ingredients were fed to the birds in the form of mesh and by hand method. Hydroxy‐selno‐methionine was also added to the diets in powder form.

### 2.2 Performance Parameters

The number of eggs produced, egg weight and mortality were checked daily during the experimental period. Egg mass (grams per hen per day) and FCR were calculated weekly. The egg production percentage was evaluated by dividing the total number of eggs produced in each repelication by the hen's day. The egg mass was estimated by multiplying the average egg weight of each replicate by the percentage of egg production. In addition, weekly feed intake was divided by egg mass to estimate FCR.

### 2.3 Reproductive performance

Once every 21 days, the number of settable eggs, percentage of hatchability percentage, percentage of fertile eggs, embryonic losses (percentage of fertilized eggs) and percentage of grade one chicks were checked.

Fertility percentage was checked using nose light (candling) on the 12th day after laying in the incubator. The hatched chicks were divided into grades one and two according to freshness, umbilical cord condition, movement problems and other appearance characteristics.

### 2.4 Sperm quality parameters

#### 2.4.1 Semen collection

A common abdominal massage technique (Mosayyeb Zadeh et al., 2023) was used to collect seminal fluid samples from the roosters at the end of the experiment (week 60). (Mosayyeb Zadeh et al., 2023). Moreover, it was attempted to immediately reach the obtained semen samples at the Reproductive Physiology Labratory of Urmia University (less than 10 min) to mixed them with the provided poultry extender (PSE) for further examinations. The PSE was prepared according to a method disribed by Wilcox et al. (1961) with slight modifications.

#### 2.4.2 Sperm motility

Total motility, progressive motility, average velocity along the path, velocity along the straight path, velocity along the curved path and the linearity of the motility by a computer analysis system equipped with a phase contrast microscope (LABOMED, model LBO9126017T, Lx 400; Labomed America Inc.) were evaluated with 100 magnification and CASA software (Sperm 3.2 VideoTest). For this purpose, 10 µL of the diluted semen sample were poured on the slide a clean slide was placed on it, and the sperm parameters were evaluated using a computer (da Silva Maia et al., 2009).

#### 2.4.3 Plasma membrane integrity

This test was designed based on the hypoosmotic swelling test containing fructose (9 g) and sodium citrate (4.9 g), 1000 mL of distilled water with an osmolarity of 100 mOsm in the sperm medium. Spermatozoa with a knotted tail were considered spermatozoa with an integral membrane, and spermatozoa with a straight tail were considered spermatozoa with a non‐integral membrane (Revell & Mrode, 1994). For this purpose, 10 µL of diluted sperm were mixed with 20 µL of host solutions, and the state of sperms with smooth tail and complex tail was examined and evaluated under a microscope (Olympus) with 100× magnification.

#### 2.4.4 Sperm viability

The viability of sperm was checked by eosin–nigrosin staining. For this purpose, 10 µL of diluted semen were mixed with 20 µL of dye containing eosin dye (1.67 g), nigrosin dye (10 g) and sodium citrate (2.9 g) in 100 mL of distilled water. It was prepared on a glass slide and the survival of 200 sperms was examined using a light microscope (Olympus) with 100× magnification. Sperms that completely or partially took on a reddish‐purple colour were considered dead, and sperms that did not acquire colour were considered live sperms (Khalil‐Khalili et al., 2021).

#### 2.4.5 Sperm abnormality

Sperm abnormality was also evaluated by the eosin–nigrosin staining method as described above (Khalil‐Khalili et al., 2021). After drying, slides were evaluated with 100× magnification. In this way, by counting 200 sperms per slide, sperms with twisted tails, double tails, abnormal tails, tailless heads and double heads were considered abnormal spermatozoa.

#### 2.4.6 Semen malondialdehyde

The procedure used for the semen malondialdehyde (MDA) determination assay involved reacting the sample MDA with thiobarbituric acid, extracting the sample with butanol‐normal, and comparing the light absorbance curve obtained through spectrophotometry with the standard. This was accomplished by first dissolving 500 µL of the semen sample in 3 mL of 1% phosphoric acid, vortexing the mixture, and then adding 1 mL of 0.675% thiobarbituric acid and vortexing it once more. The resultant mix spent 45 min in a boiling water bath. Subsequently, 3 mL of 1% butanol normal was added, vortexed for 1–2 min, and centrifuged at 3000 rpm for 10 min. In addition, the upper phase was separated, and their light absorbance (vs. blank [butanol normal]) was read at a wavelength of 532 nm. Eventually, the semen MDA was determined by comparing the light absorbance of the sample with that of the standard. Notably, the MDA standard (in six concentrations: 0.5, 1, 2, 4, 8 and 12 microliter) was prepared by dissolving 1,1,3,3‐tetramethoxy propane (C7H16O4) in deionized water.

### 2.5 Statistical analysis

Data normality was tested with the UNIVARIATE PROC and Shapiro–Wilk test in SAS 9.4. MIXED PROC (SAS, 2003) was used to analyse repeated measurement data (productive and reproductive performance), and GLM PROC was used for egg quality. Means were compared with the Tukey multiple range test at the 5% level (Table 1).

## 3 RESULTS

### 3.1 Productive performance

Table 2 shows the effect of different experimental treatments on the production performance of broiler breeders. The results showed that egg production, weight, mass and FCR are affected by treatment, time and the interaction effect of treatment and time. The use of OH‐SeMet in the diet of broiler breeders and the diet of both hens and roosters improved the laying percentage, egg weight, egg mass and FCR compared to the <span>HSM </span> control treatment (*p* < 0.05). Moreover, feeding both hens and roosters with a diet containing OH‐SeMet improved the mentioned parameters compared to feeding broiler breeders alone (*p* < 0.05).

**TABLE 2:** Effects of hydroxy‐selenomethionine on the production performance of broiler breeders (Ross 308).

Item		Egg production (%)	Egg weight (g)	Egg mass (g)	FCR
Treatments^1^	T1	68.86^d^	66.41^c^	45.56^c^	3.50^a^
	T2	69.55^c^	66.38^c^	45.97^c^	3.46^b^
	T3	70.98^b^	67.90^b^	48.08^b^	3.31^c^
	T4	72.99^a^	68.23^a^	49.66^a^	3.20^d^
SEM		0.18	0.05	0.11	0.01
*p*‐Value	Treat	<0.001	<0.001	<0.001	<0.001
	Time	<0.001	<0.001	<0.001	<0.001
	Treat × Time	<0.001	<0.001	<0.001	<0.001

*Note*: Means within the same column with different letters (a–d) differ significantly (*p* < 0.05).

Abbreviation: SEM, standard error of means.

^1^ Experimental treatments include (1) a basal diet without OH‐SeMet (T1:control), (2) a chicken diet without OH‐SeMet and a rooster diet containing 0.1 mg/kg OH‐SeMet (T2), (3) chicken diet containing 0.1 mg/kg OH‐SeMet and rooster diet without OH‐SeMet (T3) and (4) chicken and rooster diet contained 0.1 mg/kg OH‐SeMet (T4).

### 3.2 Reproductive performance

The Effect of OH‐SeMet in the diet on the reproductive performance of broiler breeders is reported in Table 3. According to the results obtained, it has been determined that the percentage of fertile eggs, hatchability, embryonic losses and grade one chicks are affected by treatment and time. The interaction of treatment and time affected fertility percentage, hatchability and embryonic losses. The fertility and hatchability percentage of T4 and T2 treatments increased fertile and hatchability percentages compared to control and T3 treatments (*p* < 0.05). The percentage of embryonic losses in cotrol was higher than in other treatments, but grade one chickens were higher in T4 than in other treatments (*p* < 0.05).

**TABLE 3:** Effects of hydroxy‐selenomethionine on the reproductive performance of broiler breeders (Ross 308).

Item		Settable eggs (%)	Fertile egg (%)	Hatchability of settable egg (%)	Embryonic losses of settable egg (%)	Grade one chicks (%)
Treatments^1^	T1	91.58	92.14^c^	83.95^c^	9.15^a^	97.98^b^
	T2	90.16	95.06^a^	86.85^a^	8.21^b^	98.01^b^
	T3	90.41	93.29^b^	85.45^b^	7.83^b^	98.10^b^
	T4	88.37	96.38^a^	87.23^a^	8.18^b^	98.32^a^
SEM		0.91	0.14	0.17	0.15	0.06
*p*‐Value	Treat	0.06	<0.001	<0.001	<0.001	0.003
	Time	<0.001	<0.001	<0.001	<0.001	<0.001
	Treat × Time	0.87	<0.001	0.02	0.01	0.08

*Note*: Means within same column with different letters (a–c) differ significantly (*p* < 0.05).

Abbreviation: SEM, standard error of means.

^1^ Experimental treatments include: (1) a basal diet without OH‐SeMet (T1:control), (2) a chicken diet without OH‐SeMet and a rooster diet containing 0.1 mg/kg OH‐SeMet (T2), (3) chicken diet containing 0.1 mg/kg OH‐SeMet and rooster diet without OH‐SeMet (T3) and (4) chicken and rooster diet contained 0.1 mg/kg OH‐SeMet (T4).

### 3.3 Sperm quality parameters

**FIGURE 1:**
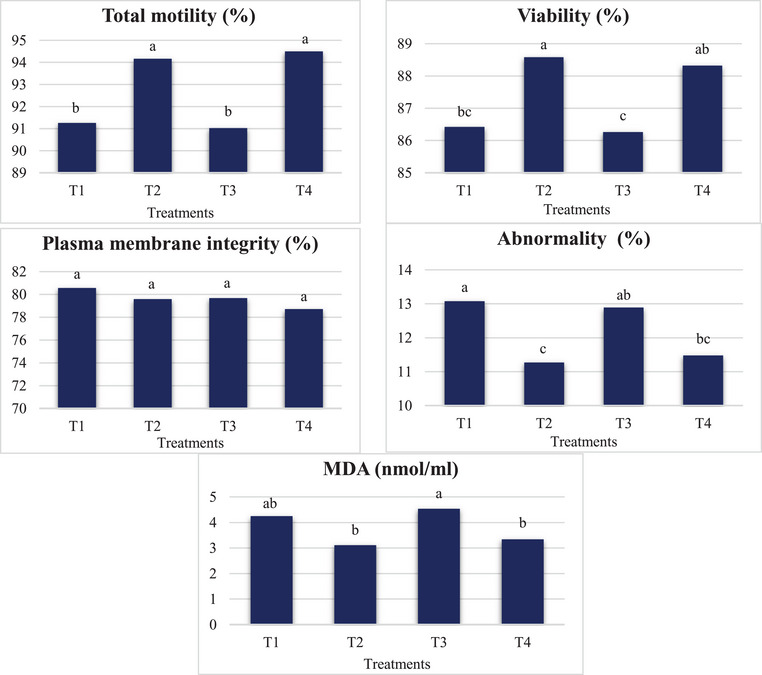
Effects of hydroxy‐selenomethionine on the sperm quality parameters of broiler breeder roosters (Ross 308). Experimental treatments include: (1) a basal diet without OH‐SeMet (T1: control); (2) a chicken diet without OH‐SeMet and a rooster diet containing 0.1 mg/kg OH‐SeMet (T2); (3) chicken diet containing 0.1 mg/kg OH‐SeMet and rooster diet without OH‐SeMet (T3) and (4) chicken and rooster diet contained 0.1 mg/kg OH‐SeMet (T4). MDA, Malondialdehyde.

**FIGURE 2:**
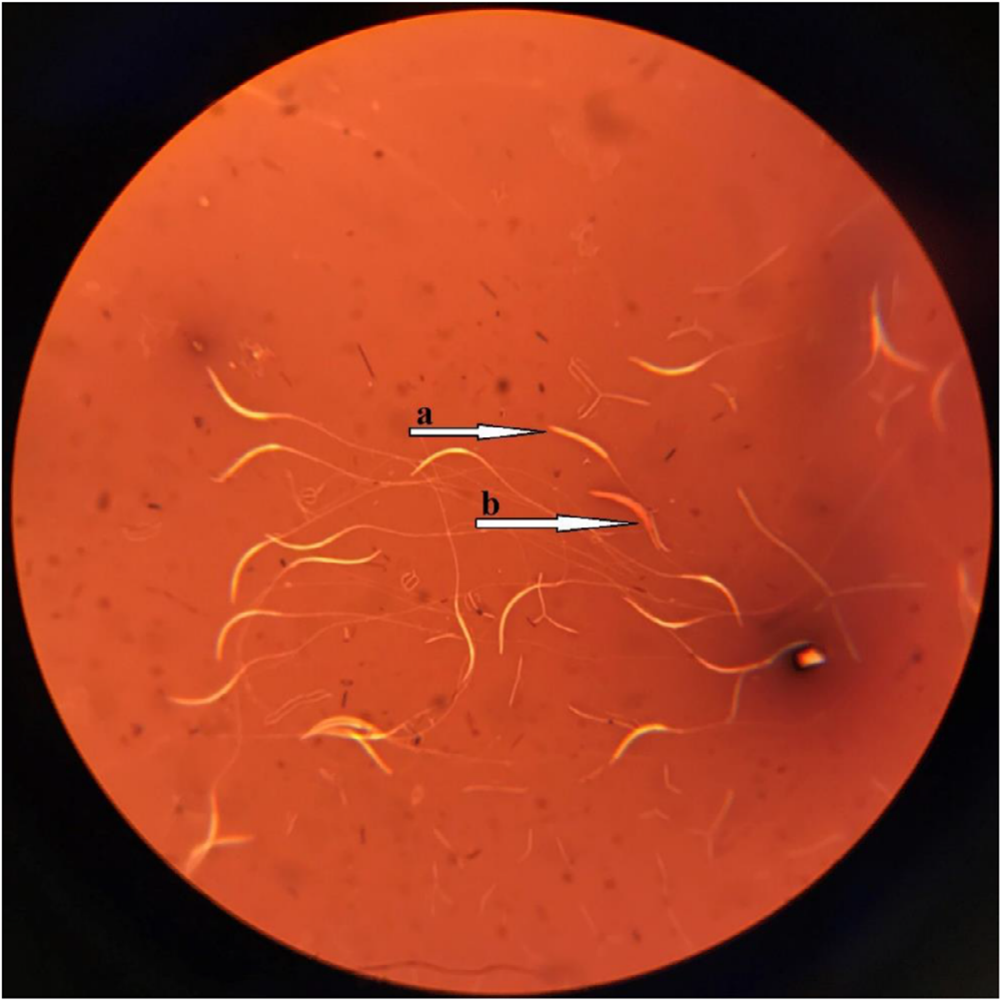
A light micrograph of the eosin–nigrosin‐stained sperms. The stained and unstained sperms were considered dead and live sperms, respectively. The black arrows illustrate examples of: (a) alive/not damaged and (b) dead/membrane damaged sperm.

The results related to the effect of OH‐SeMet in the diet on the qualitative parameters of the sperm of broiler breeder roosters are given in Table 4. Statistical analysis showed that total motility, viability, abnormality (Figures 1 and 2) and MDA concentration were affected by OH‐SeMet. Total mobility and viability were significantly higher in T2 and T4 treatments than in control and T3 treatments. In the T2 and T4 treatments, sperm abnormality percentage and sperm MDA concentration were decreased.

**TABLE 4:** Effects of hydroxy‐selenomethionine on the sperm quality parameters of broiler breeder roosters (Ross 308).

Item		Total motility (%)	Viability (%)	Plasma membrane integrity (%)	Abnormality (%)	MDA (nmol/mL)
Treatments^1^	T1	91.25^b^	86.42^bc^	80.56	13.08^a^	4.25^ab^
	T2	94.17^a^	88.58^a^	79.58	11.27^c^	3.11^b^
	T3	91.03^b^	86.26^c^	79.66	12.89^ab^	4.54^a^
	T4	94.50^a^	88.32^ab^	78.71	11.48^bc^	3.34^b^
SEM		0.55	1.08	1.84	0.79	0.65
*p*‐value		<0.001	0.005	0.48	0.003	0.007

*Note*: Means within same column with different letters (a–c) differ significantly (*p* < 0.05).

Abbreviations: MDA, malondialdehyde; SEM, standard error of means.

^1^ Experimental treatments include: (1) a basal diet without OH‐SeMet (T1: control), (2) a chicken diet without OH‐SeMet and a rooster diet containing 0.1 mg/kg OH‐SeMet (T2), (3) chicken diet containing 0.1 mg/kg OH‐SeMet and rooster diet without OH‐SeMet (T3) and (4) chicken and rooster diet contained 0.1 mg/kg OH‐SeMet (T4).

## 4 DISCUSSION

Broiler breeders gradually decrease productivity as their age increases, and the proportion of egg production declines (Dunn, 2013). Ovarian aging causes a sharp decrease in the egg production of broiler breeders after 400–480 days, which lowers the breeders’ commercial worth and egg production. The current study found that adding 0.1 mg/kg of OH‐SeMet to the diet of broiler breeders increased egg laying, egg weight and egg mass and decreased feed conversion ratio (FCR) more effectively than control. Studies have shown that selenium has positive effects on broiler breeders’ productivity. When compared to inorganic Se, feeding organic Se to broiler breeders can reduce mortality and increase egg production (Rajashree et al., 2014). Li et al. (2024) results revealed than SM increased the laying rate compare to sodium selenite, whereas different Se levels had no effect. Some studies on the impact of Se sources on poultry performance have produced contradictory findings. According to Liu et al. (2020), diets with varying Se sources and concentrations significantly impacted daily feed intake and egg production rate but did not impact mean egg weight or FCR. However, no variation was also found in the impact of Se sources on production performance (Dalia et al., 2017; Woods et al., 2020). Variations in the results could be caused by factors including the type of bird, diet and environment, amount of Se added, source and length of experiment. Se is a necessary component of deiodinase, which helps maintain thyroid hormone levels through metabolism (Daniels, 1996). Wang et al. (2021) applied L‐selenomethionine instead of sodium selenite and reported a significant increase in the mRNA level of deiodinase‐1 in the liver of 1‐day‐old chickens. This suggests that using organic Se can better activate thyroid hormones, improve protein digestibility, and improve energy metabolism compared to inorganic forms (Saleh, 2014). Another potential reason is the high bioavailability of OH‐SeMet, as the chemical structure of OH‐SeMet is very similar to methionine, and Met‐tRNA cannot distinguish between methionine and OH‐SeMet. This allows OH‐SeMet to combine directly with proteins in the body, and be used as a source of Se (Hachemi et al., 2023). It has been suggested that higher expression of selenoprotein may better demonstrate the nutritional status and metabolic function of Se (Wang et al., 2022). Li et al. (2024) results showed that SeMet supplementation led to increased mRNA expression of most selenoproteins in liver, suggesting that supplementation with organic Se improves Se utilization. Selenoprotein is essential for calcium flux in immune cells, T‐cell proliferation, and neutrophil migration (Zoidis et al., 2018) and is involved in the translocation of misfolded proteins in the endoplasmic reticulum (Curran et al., 2005). Ultimately, these mechanisms could lead to improvements in production the performance of old broiler breeders.

The nutrients found in breeder eggs are derived from broiler breeders’ diet and metabolic activities. The hatching rate and the embryos’ health are significantly affected by the nutrients (including vitamins and trace elements) that broiler breeders absorb, metabolize and deposit. One efficient method of raising the Se content in eggs is to take dietary supplements (Surai & Fisinin, 2014). This is potentially due to the higher levels of Se deposited in broiler breeders' eggs, which also found that adding OH‐SeMet to the diet greatly increased the hatching rate and decreased embryo losses (Emamverdi et al., 2019). According to reports, the conventional method of selenite application fails to cross the maternal blastoderm barrier effectively and results in low deposition efficiency in the eggs of broiler breeders (Wang et al., 2021). Compounds found in OH‐SeMet can efficiently pass through the foetal disc barrier via amino acid metabolism. According to Surai and Fisinin (2014), it can also be efficiently deposited in eggs and embryos, enhancing their capacity to absorb antioxidant defenses and positively impacting the hatchability and survival of their offspring. Yuan et al. (2011) found that selenomethionine increased the amount of Se in egg yolk and egg white compared to sodium selenite. This result might be explained by the distinct ways organic and inorganic Se are absorbed. Inorganic Se is absorbed passively from the intestine via a straightforward diffusion process, and it competes with numerous mineral elements for absorption pathways. On the other hand, organic Se has a higher bioavailability than inorganic form and is actively absorbed via the amino acid transport mechanism (Gammelgaard et al., 2012). Breeder eggs deposited with Se have been shown to have a strong maternal effect, increase hatching rate and embryo survival, and effectively protect embryos from free radical damage during growth and development (Surai, 2000).

The chick embryo's respiratory mode switches from chorioallantoic respiration to pulmonary respiration during the middle and end stages of egg incubation. This accelerates aerobic metabolism and causes the developing chick embryo to produce excessive oxygen free radicals. This phenomenon results from lipid peroxidation and causes harm to the chicken embryo's organs and tissue, and can lead to the embryo's eventual death (Visschedijk, 1968). Therefore, the development of chicken embryos requires a fully functional antioxidant system. According to Meng et al. (2019), Se has a major regulatory effect on the antioxidant capacity of developing embryos, offspring and broiler breeders. Se plays a dual role in expressing and synthesizing over 25 selenoproteins, including deiodinase and GSHpx.

Furthermore, Se aids in developing the body's potent antioxidant defense system by influencing both enzymatic and non‐enzymatic antioxidant defense mechanisms (Surai & Kochish, 2019). Li et al. (2020) studied the effects of different sources of Se in the diets of broiler breeders exposed to acute oxidative stress and found that supplementing with selenomethionine decreased embryo mortality. The current study demonstrates the long‐term maternal effects of organic Se supplementation by showing that adding OH‐SeMet to the diets of broiler breeders significantly increases the percentage of grade one broiler. According to recent developments in maternal programming and gene expression, the post‐natal development of chicks may be more closely correlated with maternal effects than previously thought. The maternal‐effect phenomenon may have a plausible explanation according to epigenetic mechanisms, although the underlying molecular mechanisms are still being studied (Pinney & Simmons 2012). As per Berghof et al. (2013), the phenotypic characteristics of the next generation can vary based on their mothers’ nutritional and environmental conditions. Different effects on offspring may be expected since commercial poultry are raised are in stressful and dissimilar conditions compared to those of their wild ancestors (Dixon et al., 2016).

The results of this research showed that qualitative sperm parameters were improved in roosters receiving OH‐SeMet, which may be due to the insufficient amount of Se required by roosters with the basic diet. When sperm are exposed to excessive amounts of free radicals, peroxidation of membrane lipids occurs, which is an autocatalytic self‐propagating reaction and is associated with impaired motility, viability and function of the plasma membrane of sperm. In this regard, it has been shown that free radicals have a negative effect on the motility and the percentage of sperm with a healthy plasma membrane and can reduce the quality of rooster sperm and its ability to fertilize (Raei et al., 2021). On the other hand, during the early stages of spermatogenesis, GPX4 is believed to protect developing sperm against DNA damage caused by oxidative stress. In the next stages, this selenoprotein becomes a structure of the mitochondrial sheath around the flagellum, which is necessary for the stability and motility of the sperm through cross‐linking with proteins and creating integrating in the middle part of the sperm (Přinosilová et al., 2014). Therefore, adding Se can improve sperm motility and viability. In our study, the addition of OH‐SeMet increased the percentage of motility and sperm viability of old roosters and decreased the percentage of abnormality. In line with the results of this study, it has recently been shown that supplementation of organic Se in the diet of roosters improves sperm motility and viability (Bălăceanu et al., 2022). In another study, the addition of yeast Se to the diet of broiler breeder roosters (52 weeks old) showed that this compound improves sperm motility, viability and plasma membrane integrity (Raei et al., 2021). Consistent with the results of previous studies (Alavi et al., 2020; Chauychu‐Noo et al., 2021), in the present study, it was shown that OH‐SeMet is more effective than control in improving sperm motility and viability, which is likely due to the supply of Se required by roosters. According to the findings of a preliminary study, the proportion of abnormal sperms in rats fed with a Se‐free diet was increased compared to the control group (Słowińska et al., 2011), which indicates the importance of this micronutrient in sperm production. Moreover, it has been stated that the Se concentration of seminal plasma negatively correlates with the morphological abnormality of sperm (Přinosilová et al., 2014). Most of the antioxidant undergo changes during the life of living organisms with increasing age (Lopez et al., 1994) and it has been stated that oxidative stress is the reason for the reduction of sperm quality in aged mice (Jervis & Robaire, 2004). Also, due to the old age of the rooster in the present study, oxidative stress probably led to a decrease in the level of sperm antioxidant (as it was also determined by the increase in MDA level). Therefore, low level of Se below the required threshold is a reason for the low quality of sperm characteristics in control treatment. Se deficiency leads to damage in the middle part of the sperm, reducing its motility and increasing morphological abnormalities, especially in sperm head area (Agarwal & Sekhon, 2010). The effect of antioxidant on sperm quality can be affected by dose or treatment period.This is due to the fact that supplements must reach a certain concentration to have an effect on oxidative stress (Molina et al. 2010). In the present study, the increase in sperm motility and viability in the groups containing OH‐SeMet compared to the control group can be caused by the effect of Se on the oxidative phosphorylation of sperm mitochondria and the protection of sperm DNA against oxidative stress (Kaushal & Bansal, 2007).

MDA is a reactive and mutagenic lipid peroxidation product in seminal plasma (Shang et al., 2004) and can be considered a diagnostic tool for male infertility (Tavilani et al., 2008). A high negative correlation between MDA and sperm motility has been shown in pigs (Breininger et al., 2005). In rooster sperm, low‐quality semen, such as those with low sperm motility, is associated with higher amounts of MDA (Amini et al., 2015). Peroxidation causes adverse changes in the structure of the acrosomal part of sperms and thus reduces the motility and viability of sperms (Dimitrov et al., 2007). According to Brown and Burk (1973), Se is found in the middle of sperm, and a deficiency results in abnormal morphology, decreased motility and infertility. Lower sperm MDA and higher sperm quality parameters are most likely the cause of the higher fertility rate seen in the OH‐SeMet groups. Infertile men's semen has reduced Total antioxidant capacity (TAC) and individual antioxidant levels (Lewis et al., 1995). Eroglu et al. (2014) found a positive correlation between seminal plasma TAC levels and seminal fluid quality parameters including concentration, motility and morphology in men with idiopathic infertility. The primary determinants of bird fertility are the quantity of sperm retained in the sperm storage and release tubes as well as their capacity to unite and pierce the perivitelline layer. The increased quantity and quality of sperm kept in the sperm storage tubes, which were then used in fertilization, is most likely the cause of the higher fertility rate seen in roosters fed OH‐SeMet (Zhandi et al., 2020).

## 5 CONCLUSION

In general, the results of this study showed that with 0.1 mg/kg of OH‐SeMet in the diet of old broiler breeders, egg production, egg weight, FCR and hatchability improved compared to control. Therefore, the use of OH‐SeMet may be a practical approach to help the production and reproduction performance of old broiler breeders. Supplementation of OH‐SeMet in old broiler breeders’ diets had a positive effect on the quality of hatched chickens, however, it is suggested that the performance of hatched broiler chickens should be evaluated until the age of 42 days. The addition of Se OH‐SeMet to the diet of roosters increased the percentage of sperm motility and viability and decreased the percentage of sperm abnormalities. Therefore, it can be stated that an essential diet does not fully supply the Se required by broiler breeders and roosters (Figure 1 and 2).

## AUTHOR CONTRIBUTIONS

**Ahmad Manafi**: Data curation; investigation; writing – original draft. **Yahya Ebrahimnezhad**: Conceptualization; methodology; writing – review and editing. **Habib Aghdam Shahryar**: Conceptualization; methodology; validation. **Abdolahad Shaddel Teli**: Project administration. **Abolfazl Gorbani**: Formal analysis. **Naser Maheri‐Sis**: Data curation; supervision.

## CONFLICT OF INTEREST STATEMENT

The authors declare no conflicts of interest.

## FUNDING INFORMATION

None.

## ETHICS STATEMENT

The authors confirm that the ethical policies of the journal, as noted on the journal's author guidelines page, have been adhered to and the appropriate ethical review committee approval has been received. The Iranian Council of Animal Care guidelines for the Care and Use of Experimental Animals, vol. 1. Isfahan University of Technology Isfahan, Iran were followed.

### PEER REVIEW

The peer review history for this article is available at https://publons.com/publon/10.1002/vms3.1538.

## Data Availability

The datasets used and/or analysed during the current study are available from the corresponding author on reasonable request.
